# A comparative analysis of biologics market dynamics in 12 countries: (Bio)similar and sustainability

**DOI:** 10.3389/fphar.2025.1659395

**Published:** 2025-11-11

**Authors:** Younghyun Song, Gyeongseon Shin, Gyeyoung Choi, Euna Han, Huang-tz Ou, SeungJin Bae

**Affiliations:** 1 College of Pharmacy, Ewha Womans University, Seoul, Republic of Korea; 2 Department of Pharmacy, Wonkwang University, Jeonbuk, Republic of Korea; 3 College of Pharmacy, Yonsei University, Incheon, Republic of Korea; 4 Department of Pharmacy and Institute of Clinical Pharmacy and Pharmaceutical Sciences, National Cheng Kung University, Tainan, Taiwan

**Keywords:** biosimilar, market dynamics, price index, expenditure, healthcare system sustainability

## Abstract

**Objective:**

We sought to evaluate the relationship between biosimilar introduction and the prices and expenditures of biologics in high-income countries.

**Methods:**

This study examined IQVIA-MIDAS sales data for biologics and biosimilars from January 2018 to June 2020 across 12 high-income countries (Australia, Austria, Canada, France, Germany, Italy, Japan, Korea, Spain, Sweden, Switzerland, and the United Kingdom). We selected seven biologics that fall under the World Health Organization Anatomical Therapeutic Chemical code L (Antineoplastic and immunomodulating agents), and categorized them into two groups based on the availability of biosimilar data. Group A consisted of biologics with biosimilars-etanercept, infliximab, rituximab, and trastuzumab, while Group B comprised biologics without biosimilars, including cetuximab, nivolumab, and pembrolizumab. A descriptive analysis was conducted to investigate the association between biosimilar introduction and trends in Fisher price index and relative expenditure. In addition, two-sample t-test and Wilcoxon rank-sum test were used to assess the statistical significance of differences in growth rates between the two groups.

**Results:**

Group A exhibited a declining trend in the Fisher price index, whereas Group B remained stable across most countries, with average compound quarterly growth rates being −2.19% and −0.54%, respectively (p < 0.001). Relative expenditure trends also revealed contrasting patterns between the groups across most countries, further highlighting the differences. The average compound quarterly growth rates for relative expenditures was −1.78% for Group A and 7.66% for Group B (p < 0.001).

**Conclusion:**

The introduction of biosimilars was significantly associated with reductions in the prices and expenditures of biologics in high-income countries. This underscores the potential role of biosimilars in supporting the long-term sustainability of the healthcare system.

## 1 Introduction

The expansion of the biologics market is a major driver of rising pharmaceutical expenditures (Yazdany, 2020). The annual approval rate of biologics by the U.S. Food and Drug Administration (FDA) has shown an upward trend, surpassing that of small-molecule new molecular entities (NMEs) in 2022 (Senior, 2023). In 2021, biologics accounted for four of the ten highest-selling drugs, including adalimumab, nivolumab, pembrolizumab, and Ustekinumab (Urquhart, 2022). The emergence of these high-cost biologics has substantially increased the financial burden on patients and healthcare payers (Buske et al., 2017). Annual economic burden for biologics ranges from $10,000 to $30,000, with some exceeding $50,000 (Chen et al., 2018).

The introduction of biosimilars has played a pivotal role in reshaping the pricing landscape of biologics. As patents for major biologics expire and biosimilars enter the market, competition intensifies (Carl et al., 2022; Lyman et al., 2018; Renwick et al., 2016). By fostering market competition, biosimilars have been associated with significant price reductions for both originator biologics and the biosimilars themselves (Robinson and Jarrion, 2021), ultimately promoting more sustainable pricing strategies across therapeutic classes. The resources saved from increased biosimilar adoption can be strategically reinvested within healthcare systems, helping to fund access to new, premium-priced medicines and ensuring that healthcare systems can sustainably manage both existing and emerging therapeutic needs (Moorkens et al., 2021; Povero and Pradelli, 2020). Moreover, the availability of cost-effective biosimilars can improve patient access to essential biologic therapies, particularly in settings where high drug costs have historically limited utilization (Chen et al., 2024; Peng et al., 2023; Woo et al., 2023). Thus, understanding the market dynamics associated with biosimilar introduction is critical not only for evaluating cost containment strategies but also for informing policies that promote the long-term sustainability of healthcare systems (Kim et al., 2020; Moorkens et al., 2017).

Yet, the uptake and impact of biosimilars exhibit considerable variation across countries, driven by differences in regulatory frameworks, pricing and reimbursement mechanisms, prescriber practices, and patient awareness (Mroczek et al., 2024). In the United States, adoption has been relatively limited, constrained by complex interchangeability requirements and entrenched brand loyalty among prescribers and patients (Dalpoas et al., 2020). Conversely, several European countries—particularly those with centralized procurement systems and robust policy incentives, such as the United Kingdom and Germany—have demonstrated more rapid uptake and pronounced price reductions (Moorkens et al., 2017; Rémuzat et al., 2017). In Asia, countries such as Japan and South Korea display heterogeneous patterns shaped by stringent regulatory standards and government-led cost-containment strategies (Kim et al., 2020; Mamiya et al., 2025). These cross-national differences highlight the need to contextualize biosimilar market dynamics to better understand their potential for cost savings and improved access.

However, there are limited studies examining market dynamics and trends across multiple biologics and countries. While previous studies on biologics have explored international price comparisons and market dynamics, some were outdated (Danzon and Furukawa, 2006), other focused on a limited number of molecules (Kim et al., 2020; Woo et al., 2023) or countries (Carl et al., 2022; Dean and Bond, 2021), and few addressed fluctuation in expenditure (Böhm et al., 2023; Chen et al., 2024; Pontes et al., 2024). This study aims to analyze the market dynamics of biosimilars by classifying multiple biologics based on the introduction of biosimilars, with a specific emphasis on widely used biologics that represent major therapeutic markets. The primary objective of this study is to assess whether the introduction of biosimilars is associated with price reductions and, ultimately, cost savings across 12 high-income countries in Asia, Europe, and North America.

## 2 Methods

### 2.1 Data sources

This study employed IQVIA-MIDAS data to analyze the prices and expenditures of biopharmaceutical molecules from Q1 2018 to Q2 2020. This database provides quarterly sales volume and value data of pharmaceutical products sold in hospital and retail sectors, and has been used as a data source in the previous studies (Chen et al., 2024; Kim et al., 2020; Peng et al., 2023; Woo et al., 2023). Prices were calculated by dividing the sales value by the sales volume (Kim et al., 2020; Peng et al., 2023), and sales volume was measured using the standard unit (SU), a metric commonly utilized in prior studies (Kim et al., 2020; Magazzini et al., 2004; Timur, 2011). We used sales value data reported as ‘Local Currency Dollars (LCD)’, which IQVIA-MIDAS defines as local currency sales converted to US dollars using constant exchange rates (Blais et al., 2022).

### 2.2 Country selection

For this analysis, we selected high-income countries from the Organisation for Economic Co-operation and Development (OECD) where data on both originators and biosimilars were available during the study period. To maximize comparability across jurisdictions, we focused on high income countries defined based on the World Bank country classifications by income level (World Bank Group, 2023), with broadly comparable reimbursement and procurement frameworks. Among the 34 high-income OECD members (two in North America, 26 in Europe, and six in the Asia–Pacific region), 12 countries—eight European countries (Austria, France, Germany, Italy, Spain, Sweden, Switzerland, and the United Kingdom), along with Australia, Canada, Japan, and Korea—were included, considering data availability and the proportional distribution of countries across continents. Low- and middle-income countries (LMICs) were excluded, as access to biologics—particularly for high-priced biologics and biosimilars—is constrained by economic and societal factors (Calamia and Abraham, 2023). The United States was also excluded because its distinct health-insurance system with high medical spending and relatively limited biosimilar uptake hamper comparability with other markets (Kvien et al., 2022; Schoen et al., 2010).

### 2.3 Molecule selection

We selected seven biologics—cetuximab, etanercept, infliximab, nivolumab, pembrolizumab, rituximab, and trastuzumab—that had consistent data availability for both originators and biosimilars available across all 12 study countries. These biologics fall under the L (Antineoplastic and Immunomodulating Agents) class in the World Health Organization (WHO) Anatomical Therapeutic Chemical (ATC) classification (WHO Collaborating Centre for Drug Statistics Methodology, 2023). Notably, although selection being based on data availability across study countries rather than sales ranking, all seven biologics are considered blockbuster products with annual global sales exceeding USD 1 billion, indicating their status as widely used therapies in major therapeutic markets (online Supplementary Table 1). However, combination products involving biosimilars or originators (e.g., pertuzumab/trastuzumab) were excluded. Molecules for which originator or biosimilar data were unavailable in some of the 12 countries during the study period (e.g., adalimumab, bevacizumab) were also excluded.

To ensure comparability across jurisdictions, the data were analyzed for specific dosage forms for each molecule: cetuximab 100 mg (5 mg/mL*20mL, 2 mg/mL*50 mL), etanercept 50 mg/mL*1mL, infliximab 100mg, nivolumab 10 mg/mL*10mL, pembrolizumab 25 mg/mL*4mL, rituximab 10 mg/mL*50mL, and trastuzumab 150 mg. However, some exceptions were made due to data availability constraints. In Korea, etanercept 50 mg/mL*1 mL originator entered the market in Q1 2013, whereas in most other countries, it was introduced before 2011. However, alternative doses (25 mg/mL*1mL and 50 mg/mL*0.5 mL) were already available before 2011 in Korea, making them more comparable to other countries in terms of market maturity. Therefore, these alternative doses were used for Korea to maintain consistency in the analysis. In Canada, trastuzumab originator data at the 150 mg dose was unavailable, so the 440 mg dose was used instead.

The seven biologics were categorized into two groups based on the availability of biosimilar data during the study period. Group A included etanercept, infliximab, rituximab, and trastuzumab, for which biosimilars were available in all study countries during the study period. Group B comprised cetuximab, nivolumab, and pembrolizumab, for which no biosimilars were available in any of the study countries during the study period.

### 2.4 Longitudinal analysis

Since data on all seven originators across the 12 countries became available starting from Q1 2018, a descriptive analysis was conducted for the 10 quarters from Q1 2018 to Q2 2020. Group A and cetuximab originators were launched prior to Q1 2011, while the remaining originators (nivolumab and pembrolizumab) and Group A biosimilars entered the market after Q1 2011. The market entry timing for Group A biosimilars and the two Group B originators is detailed in online Supplementary Table 2.

#### 2.4.1 Fisher Price Index Trends

Quarterly price trends for Group A and B within each country were calculated using the Fisher price index, which is the geometric mean of the Laspeyres and Paasche price indices (Jung et al., 2021). This index measures average price changes over time within the same country, using Q1 2018 as the reference quarter. The Fisher price index is widely used in price comparison studies as it accounts for both price variations and shifts in sales volumes (Jung et al., 2021; Kim et al., 2010). It measures the average price changes within a market basket of goods when product prices vary between two distinct situations (Danzon and Kim, 1998). It is especially useful for conducting comprehensive price comparisons across multiple countries or time periods by grouping various molecules together (Kim et al., 2010). To enhance transparency, we also report absolute weighted-average prices for each molecule in Q1 2018 (the reference quarter) in online Supplementary Table 3.

#### 2.4.2 Relative expenditure trends

Quarterly total expenditures for Group A and B were computed as the sum of the sales values for all products within each group and country. Total expenditures were expressed as relative values, with trends visualized for each country. Relative expenditure was benchmarked to Q1 2018 set as the baseline (1.00) and calculated using the formula for each group. Considering cross-country heterogeneity, absolute total expenditure trends are presented in online Supplementary Table 4.

$$Relative\;Expenditure=\frac{Total\;expenditure\;in\;each\;quarter}{Total\;expenditure\;in\;Q1\;2018}$$

#### 2.4.3 Compound quarterly growth rate (CQGR)

To compare changes in price and expenditure across countries over time, the Compound Quarterly Growth Rate (CQGR) was employed as an indicator. It was calculated as:

$$CQGR={\left(\frac{Fisher\;price\;index\;or\;relative\;expenditure\;in\;Q2\;2020}{Fisher\;price\;index\;or\;relative\;expenditure\;in\;Q1\;2018}\right)}^{\frac{1}{9}}-1$$

This metric represents the average quarterly growth rate over nine-quarters, providing a more detailed analysis of trends compared to the Compound Annual Growth Rate (CAGR), which is typically used to longer observation periods. Given its ability to capture short-term fluctuations more effectively, the use of CQGR was well-suited to the study’s quarterly data structure and relatively short duration. CQGR expresses the average percentage change per quarter in Fisher price indices and relative expenditures and was used to contextualize implications for affordability and health-system sustainability.

#### 2.4.4 Supplementary analysis

As supplementary analyses, we examined two sets of factors that could influence the primary results: (i) Group A market-share trends and (ii) biosimilar-relevant national policies across 12 countries. Market share for Group A originators and biosimilars—used as a proxy for uptake from a patient-access perspective—was calculated from sales volume measured in standard units. Policy information was compiled from prior studies and related references and categorized as supply- or demand-side. Building on previous study (Shin et al., 2025), which covered eight countries, four additional countries were added to extend the existing table. For each country, the timing of implementation was recorded for policies such as price linkage, tendering, financial incentives, prescribing guidelines, prescribing quotas, budget targets, and information/education initiatives.

### 2.5 Statistical analysis

To assess statistically significant differences in CQGR of the Fisher price index and relative expenditure trends between Group A and B, a two-tailed, two-sample t-test or Wilcoxon rank-sum test was performed. The normality of CQGR data was evaluated using the Shapiro-Wilk test. If the normality assumption was met, a two-sample t-test was applied; otherwise, the non-parametric Wilcoxon rank-sum test was used instead. We employed two-way fixed effects regression models with country and quarter fixed effects, controlling for biosimilar availability (Group A), therapeutic demand (total SU), and national policy indicators (price linkage, tendering, prescribing quota, education). The national policy indicators were defined as timing of implementation of supply- and demand-side policies for biosimilars across 12 countries, based on information from prior studies and relevant references (online Supplementary Table 5). Cluster-robust standard errors were calculated at the country level. Under two-way fixed effects, policy indicators that did not vary within countries or that shifted uniformly across all countries in the same quarter were not separately identified and were omitted due to collinearity (automatically dropped by the estimator). We report full candidate policies and indicate which were identified in the model. All statistical analyses were conducted using R version 4.3.3, with a significance level set at 0.05. Calculation for the Fisher price index, relative expenditure, and CQGR calculations were carried out using Microsoft Excel Professional Plus 2019.

## 3 Results

**FIGURE 1:**
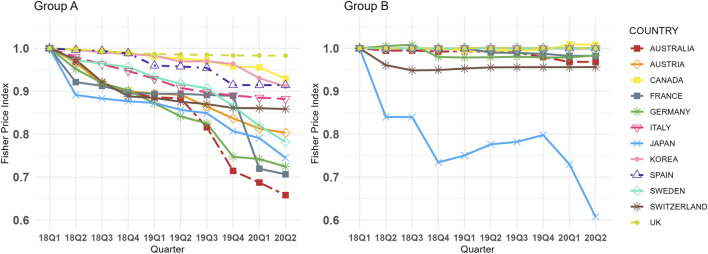
Group **(A,B)** Fisher Price Index Trends in 12 Countries (Q1 2018 in each country = 1.00, selected dosage form only). Group A = Molecules for which biosimilar data were available during the study period (etanercept, infliximab, rituximab, and trastuzumab), Group B = Molecules for which biosimilar data were not available during the study period (cetuximab, nivolumab, and pembrolizumab), selected dosage form included cetuximab 100 mg (5 mg/mL*20mL, 2 mg/mL*50 mL), etanercept 50 mg/mL*1 mL (Korea 25 mg/mL*1mL, 50 mg/mL*0.5 mL), infliximab 100mg, nivolumab 10 mg/mL*10mL, pembrolizumab 25 mg/mL*4mL, rituximab 10 mg/mL*50mL, and trastuzumab 150 mg (Canada 440 mg), absolute weighted-average prices for each molecule in Q1 2018 are presented in online Supplementary Table 3.

Figure 1 illustrates the quarterly trends in the Fisher price index for Group A and B across 12 countries from Q1 2018 to Q2 2020. During the study period, Group A generally exhibited a declining trend in most countries, whereas Group B, which lacked biosimilars, remained relatively stable except in Japan. Specifically, the CQGR of the Fisher price index for Group A ranged from −4.54% (Australia) to −0.19% (United Kingdom), while for Group B it ranged from −5.39% (Japan) to 0.09% (Canada). Notably, Japan displayed a significant downward trend in Group B. The largest absolute difference in CQGR between the two groups was observed in Australia (4.19%), while the smallest was in the United Kingdom (0.19%). On average the CQGRs between the two groups were significantly different, with Group A at −2.19% and Group B at −0.54% (p < 0.001, online Supplementary Table 6). When excluding Japan, identified as an outlier, the average CQGR for Group B adjusts to −0.10%, further widening the gap with Group A. In all countries except Japan, Group A exhibited a more negative CQGR value than Group B. Detailed Fisher price index values and CQGRs are presented in online Supplementary Table 6.

**FIGURE 2:**
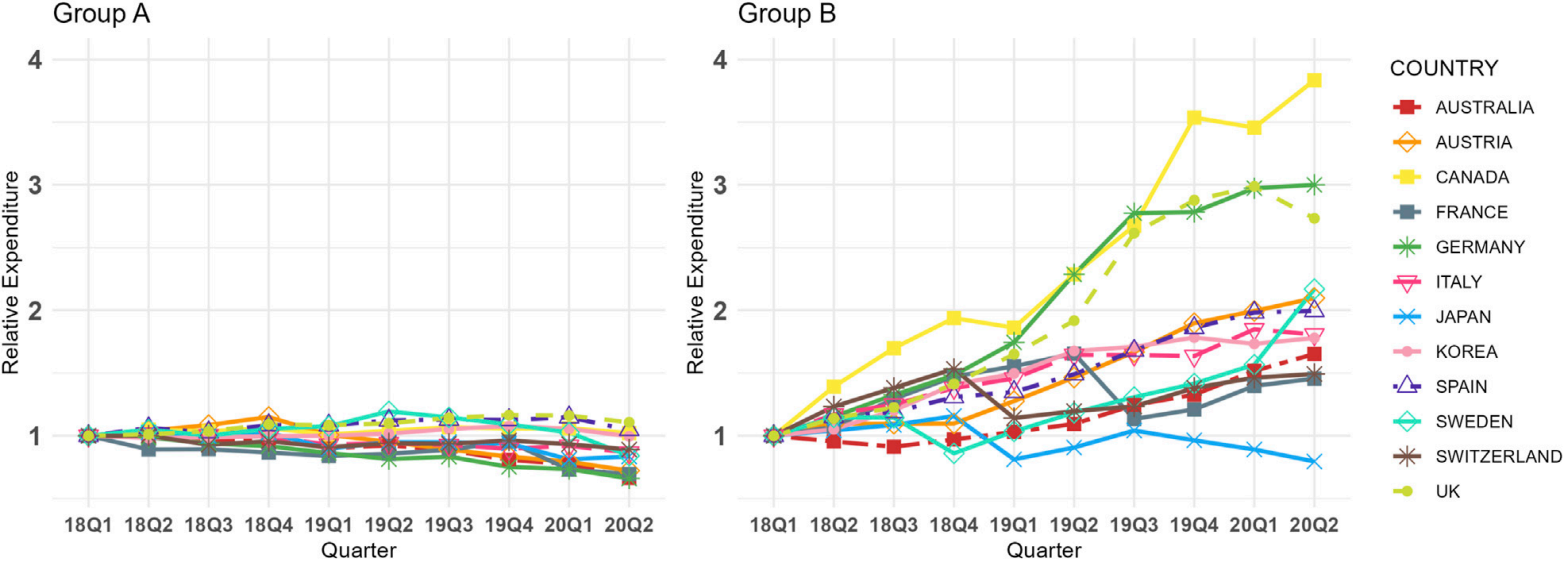
Group **(A,B)** Relative Expenditure Trends in 12 Countries (Q1 2018 in each country = 1.00, selected dosage form only). Group A = Molecules for which biosimilar data were available during the study period (etanercept, infliximab, rituximab, and trastuzumab), Group B = Molecules for which biosimilar data were not available during the study period (cetuximab, nivolumab, and pembrolizumab), selected dosage form included cetuximab 100 mg (5 mg/mL*20mL, 2 mg/mL*50 mL), etanercept 50 mg/mL*1 mL (Korea 25 mg/mL*1mL, 50 mg/mL*0.5 mL), infliximab 100mg, nivolumab 10 mg/mL*10mL, pembrolizumab 25 mg/mL*4mL, rituximab 10 mg/mL*50mL, and trastuzumab 150 mg (Canada 440 mg), absolute total expenditure trends are presented in online Supplementary Table 4.

Figure 2 shows the relative expenditure trends for Group A and B across 12 countries from Q1 2018 to Q2 2020. Similar to the Fisher price index trends, Group A generally exhibited stable or downward trends in relative expenditure across most countries, whereas Group B displayed increasing trends in all countries except Japan. These patterns align with the CQGR results, as shown in online Supplementary Table 7. Specifically, the CQGR for Group A ranged from −4.52% (Germany) to 1.14% (United Kingdom), while for Group B, it ranged from −2.52% (Japan) to 16.11% (Canada). Statistically significant differences were observed in the average CQGR between the two groups, with Group A at −1.78% and Group B at 7.66% (p < 0.001, online Supplementary Table 7). In all countries except Japan, Group A had a lower relative expenditure CQGRs than Group B, with absolute differences varying widely among countries, ranging from 0.54% (Japan) to 17.50% (Germany).

**FIGURE 3:**
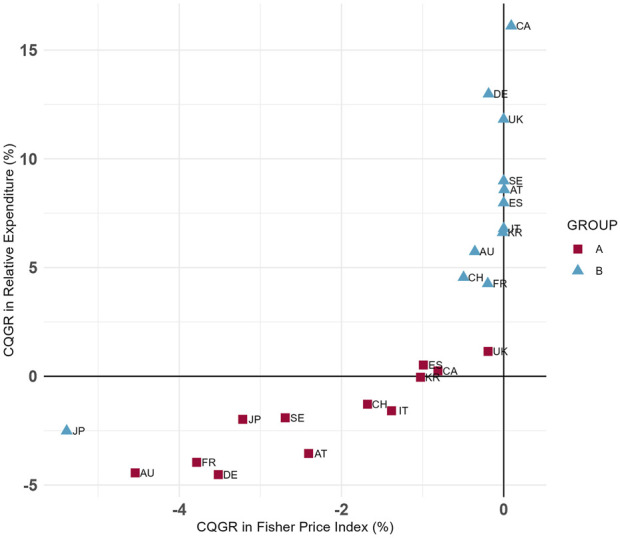
Group **(A,B)** CQGRs in 12 Countries (Selected dosage form only). Group A = Molecules for which biosimilar data were available during the study period (etanercept, infliximab, rituximab, and trastuzumab), Group B = Molecules for which biosimilar data were not available during the study period (cetuximab, nivolumab, and pembrolizumab), selected dosage form included cetuximab 100 mg (5 mg/mL*20mL, 2 mg/mL*50 mL), etanercept 50 mg/mL*1 mL (Korea 25 mg/mL*1mL, 50 mg/mL*0.5 mL), infliximab 100mg, nivolumab 10 mg/mL*10mL, pembrolizumab 25 mg/mL*4mL, rituximab 10 mg/mL*50mL, and trastuzumab 150 mg (Canada 440 mg).

Figure 3 presents the CQGRs for the Fisher price index and relative expenditures across the two groups. The plot shows that, with the exception of Japan, most countries had a lower CQGR in Group A compared to Group B. Among the countries analyzed, Australia and France exhibited some of the lowest CQGRs in both groups, while Canada and the United Kingdom recorded some of the highest values.

To further address potential confounding factors, we conducted two-way fixed effects regression models with country and quarter fixed effects. Online Supplementary Table 8 presents the results, showing that biosimilar entry (Group A) was associated with a 36% reduction in relative expenditure (p < 0.01). Policy variables showed limited independent effects, and interaction terms were not statistically significant.

In addition, we examined market-share trends for Group A originators and biosimilars as a proxy for biosimilar uptake. Although market-share levels vary across countries, all showed declining originator shares and rising biosimilar shares over time (online Supplementary Figure 1). The timing of supply- and demand-side policy implementation across the 12 countries is summarized in online Supplementary Table 5.

## 4 Discussion

Our findings suggest a strong association between the availability of biosimilars and reductions in biologic prices and expenditures. In most countries, Group A demonstrated a downward trend in both the Fisher price index and relative expenditures. In contrast, Group B maintained a stable Fisher price index while exhibited an upward trend in relative expenditures. Statistical analysis of the CQGR further confirmed a significant difference between the two groups, highlighting the crucial role of biosimilars in enhancing affordability and suggesting potential contribution to the long-term sustainability of the healthcare systems (Carl et al., 2022; Kim et al., 2020).

The impact of biosimilars on market dynamics and healthcare expenditure has been extensively explored in prior research. For instance, studies on infliximab have shown that the introduction of biosimilars led to price reduction for biologics in Australia, France, and the United Kingdom. Despite increased sales volumes as biosimilars gained market share, sales values in France and the United Kingdom remained stable (Kim et al., 2020; Pontes et al., 2024). Additionally, a study encompassing 57 countries, including the 12 countries analyzed in this study, found statistically significant price reductions for infliximab and trastuzumab following biosimilar introduction (Chen et al., 2024). The findings of this study align with the trends observed in prior research. This study expands on previous research by directly comparing the market dynamics of biologics with available biosimilars to those without. Furthermore, prior studies also show that key molecules not included in this study (e.g., adalimumab, bevacizumab) experience price reductions and expenditure declines following biosimilar entry (Alhaja and Tsourounis, 2024; Car et al., 2023; Moorkens et al., 2021; Woo et al., 2023). Taken together, this evidence suggests that expanding the analysis to additional molecules would yield consistent results and further underscores the role of biosimilars in curbing expenditures associated with high-cost biologics.

Unlike previous studies, this research offers a comprehensive analysis of the association between biosimilar introduction and biologics market dynamics. Earlier studies primarily focused on individual molecules, making it difficult to assess the broader influence of biosimilars across multiple molecules (Chen et al., 2024; Kim et al., 2020; Pontes et al., 2024; Woo et al., 2023). Moreover, prior research primarily examined price and consumption trends, often overlooking the broader financial implications for healthcare systems (Chen et al., 2024; Peng et al., 2023; Pontes et al., 2024). This study, however, addresses these gaps by integrating data across multiple molecules using price indices and CQGR, while also examining relative expenditure trends. In addition, this study compares within-country trends between groups with and without biosimilars, as pre–post analyses were not feasible for individual molecules. However, our study includes heterogeneous molecules and policies across different timeframes, which is a clear limitation. Because such heterogeneity can influence the observed market dynamics, the analysis applies two-way fixed effects (TWFE) regression, linking biosimilar entry to market dynamics and reducing bias from cross-country heterogeneity. Prior evidence shows that health-system design and policy strongly shape biosimilar penetration and price competition and indicates that policy implementation should account for product life-cycle specifics (Alnaqbi et al., 2023). The results indicate that the introduction of biosimilar is associated with reduced biologics expenditures in many high-income countries, regardless of the variations in demand- and supply-side policies. Ultimately, biosimilar can provide more affordable treatment options—particularly for patients burdened by the high costs of originator biologics—thereby improving patient access. Consistent with this, all countries in our sample showed increasing market share of biosimilars in Group A over time (online Supplementary Figure 1).

Maximizing price and expenditure reductions from biosimilars requires the implementation of effective price regulations and policies that encourage their adoption (Moorkens et al., 2016). To further explore this, we conducted supplementary two-way fixed effects regressions (online Supplementary Table 8), which showed that biosimilar entry was associated with a 36% reduction in relative expenditure (p < 0.01), whereas policy variables and their interactions demonstrated limited independent effects within the short observation window. To contextualize these findings, we further examined major policies influencing biosimilar market dynamics, summarized in online Supplementary Table 5.

Price reductions can occur through two main pathways: mandatory reductions, such as price-link policies, and voluntary reductions, where originator manufacturers lower prices to remain competitive after biosimilar entry (Renwick et al., 2016). Among the 12 countries analyzed, eight have implemented price-linkage policies. For example, Australia, Austria, and France—which exhibited the large price reductions in our analysis—apply linkage rules to both originators and biosimilars (Alnaqbi et al., 2023; Carl et al., 2022; Gregory et al., 2020; Kim et al., 2020; Machado et al., 2024; Moorkens et al., 2017; Vogler et al., 2021). By contrast, Canada and the United Kingdom, which rely on voluntary adjustments rather than formal linkage, showed the most modest decrease in Group A’s Fisher price indices during the observation period. An exception was observed in Group B, specifically in Japan, where the Fisher price index showed a more pronounced decline. This was likely due to emergency price reductions for nivolumab implemented in 2017 and 2018 (Kurokawa, 2023). In response to the high initial prices and expanded indications, the Japanese government enacted substantial price cuts, with reductions of 23.8% in April 2018 and 37.5% in November 2018 (Kurokawa, 2023). Although tender contracts are generally confidential and not publicly accessible (Renwick et al., 2016), tendering may contribute to price reductions. Australia—an early adopter of tendering (Power, 2013)—exhibited substantial price reductions. By contrast, Canada showed smaller reductions in Group A and a slight increase in Group B, consistent with the absence of a national tendering mechanism during the study period (Alnaqbi et al., 2023).

On the demand side, policies such as financial incentives, prescribing quotas, budget targets, information and education initiatives, and clinical prescribing guidelines can reduce expenditures and accelerate biosimilar uptake. Germany, for instance, exhibited the largest decline in relative expenditures, consistent with its extensive use of prescribing quotas for infliximab, etanercept, rituximab, and trastuzumab (Birkner and Blankart, 2022; Moorkens et al., 2020; Vogler et al., 2021). Prior studies confirm that these quotas have both the potential and realized effects on expanding biosimilar uptake.

The product life cycle plays an important role in pharmaceutical market dynamics. Group A consists of older molecules with earlier market entry, often facing originator market contraction due to patent expirations (Gronde et al., 2017). However, the introduction of biosimilars within this group can drive market expansion by improving patient access, as evidenced by increased overall consumption in prior studies (Peng et al., 2023; Tachkov et al., 2021; Wu et al., 2024). In contrast, Group B comprises newer compounds that are still in the growth phase, with originators yet to reach patent expiration. For multi-indication drugs, such as nivolumab and pembrolizumab, ongoing indication expansions have contributed to rising consumption. However, some countries have implemented price adjustment mechanisms during indication expansions, potentially leading to price reductions even prior to the biosimilar entry. Although product life cycles differ, each group has its own factors that can drive price reductions and market expansion. In light of these group-specific mechanisms, the results should be interpreted with caution, and differences between groups should not be attributed solely to life-cycle stage.

There are several limitations to consider. First, prices were calculated using IQVIA-MIDAS sales data, rather than directly obtaining drug prices from each country. Since IQVIA-MIDAS sales data are estimated from standardized list prices, they do not reflect actual net prices incorporating confidential agreements or discounts (e.g., tendering, managed-entry agreements; Mulcahy et al., 2024). To address data limitations, we analyzed within-country trends and relative changes over time, comparing these patterns across countries rather than focusing on absolute between-country price levels, which may be confounded by undisclosed discounts or confidential agreements. However, within-country trends may be overestimated, as prior research in five European countries included in our analysis found that net-price expenditure growth was lower than growth based on list prices (Espin et al., 2018).

Second, although this study included a larger number of countries than previous research, it focused exclusively on high-income countries. Therefore, caution should be exercised when generalizing the findings to middle- and low-income countries and the United States. To maximize the number of study countries while including products with and without biosimilars throughout the study period, our choices were inevitably constrained. Consequently, the analysis is based on seven relatively older biologics, which may narrow the analysis scope and not fully capture certain uptake patterns; thus, attention should be paid when generalizing our findings to other biologics. To demonstrate the role of biosimilars in supporting healthcare system sustainability, future studies should incorporate braoder therapies and actual treatment rates. Additionally, the relatively short observation window may not fully capture market dynamics following biosimilar entry. Because several biosimilars in Group A entered before Q1 2018, earlier price reductions and expenditure declines were outside the study window and thus not captured. In the United Kingdom, for example, Group A biosimilars already accounted for over 75% market share in Q1 2018 (online Supplementary Figure 1), indicating that substantial uptake had occurred prior to the observation period. Nevertheless, this study was designed to focus on short-term, early-adoption effects. Since prior research indicates that price and expenditure erosion typically intensify over time, the budget-saving potential of biosimilars may be greater in the longer term (Böhm et al., 2023). Thus, our estimates are likely to be conservative. To further validate the results and interpretations, future research should include LMICs and the U.S. with a broader range of biologics over an extended period.

Third, although the timing of policy implementation was examined across countries, variation in implementation details (e.g., scope, intensity, enforcement) was not incorporated into the analysis. Behavioral factors (e.g., physician and patient perceptions) that may influence biosimilar adoption and market dynamics were also not discussed. (Choi et al., 2025; Moorkens et al., 2016; Shin et al., 2024). Future research should explore how these policies, regulatory, and behavioral elements interact to shape uptake and pricing outcomes across different settings.

## 5 Conclusion

In conclusion, our analysis demonstrated that molecules with available biosimilars exhibited a decline in relative expenditure trends, whereas those without biosimilars showed an opposite trend. These findings underscore the critical role of biosimilars in enhancing affordability and their potential to support the long-term sustainability of healthcare systems.

## Data Availability

The data analyzed in this study is subject to the following licenses/restrictions: The datasets generated for this study will not be made publicly available; IMS-MIDAS can be purchased and accessed at IQVIA. Requests to access these datasets should be directed to https://www.iqvia.com.
