# Supplementary material for: A comparative analysis of biologics market dynamics in 12 countries: (Bio)similar and sustainability
Source: Front Pharmacol. 2025 Nov 11;16:1659395. doi: 10.3389/fphar.2025.1659395 (PMC12643863; doi:10.3389/fphar.2025.1659395)
Supplement: Supplementary file 1 [file DataSheet1.docx]

**Supplementary Table 1.** Annual global sales for the selected molecules.

| **Product** | **2024 Sales**  **(billion USD)** | **Reference** |
| --- | --- | --- |
| **Pembrolizumab** | 29.5 | Merck Q4 and full-year 2024 financial results  (https://www.merck.com/stories/our-q4-and-full-year-2024-financial-results/) |
| **Nivolumab** | 9.3 | Bristol Myers Squibb Reports Fourth Quarter and Full-Year Financial Results for 2024  (https://www.bms.com/assets/bms/us/en-us/pdf/investor-info/doc_financials/quarterly_reports/2024/BMY-Q42024-Earnings-Press-Release.pdf) |
| **Etanercept** | 3.3 | Amgen 2024 Annual Report Letter and 10-K  (https://www.amgen.com/-/media/Themes/CorporateAffairs/amgen-com/amgen-com/downloads/investors/2024-annual-report-letter-and-10k.pdf) |
| **Infliximab** | 1.6 | Johnson and Johnson 2024 Annual Reports  (https://s203.q4cdn.com/636242992/files/doc_downloads/Annual_meeting/2025/Johnson-Johnson-2024-Annual-Report.pdf) |
| **Rituximab** | 1.7 | Roche Assets  (https://assets.roche.com/f/176343/77b6d7edaf/02-communications-appendix-tables_fy-2024-sales-results.pdf) |
| **Trastuzumab** | 1.7 | Roche Assets  (https://assets.roche.com/f/176343/77b6d7edaf/02-communications-appendix-tables_fy-2024-sales-results.pdf) |
| **Cetuximab** | 1.4 | Merck KGaA 2024 Annual Report  (https://www.emdgroup.com/en/annualreport/2024/management-report/fundamental-information-about-the-group/company-profile-and-structure/healthcare.html) |

**Supplementary Table 2.** Timing of Group A biosimilar & Group B originator market entry, number of Group A biosimilar products in IQVIA-MIDAS database, Q1 2011-Q2 2020 (selected dosage form only).

|  | **Group A Biosimilar (number of products marketed)** | | | | **Group B Originator** | |
| --- | --- | --- | --- | --- | --- | --- |
|  | **Etanercept** | **Infliximab** | **Rituximab** | **Trastuzumab** | **Nivolumab** | **Pembrolizumab** |
| **Selected dosage** | 50mg/ml*1ml (Korea 25mg/ml*1ml, 50mg/ml*0.5ml) | 100mg | 10mg/ml*50ml | 150mg (Canada 440mg) | 10mg/ml*10ml | 25mg/ml*4ml |
| **AU** | Q2 2017 (1) | Q4 2015 (2) | Q4 2019 (2) | Q3 2019 (3) | Q1 2016 | Q3 2017 |
| **AT** | Q1 2017 (2) | Q2 2015 (4) | Q4 2017 (3) | Q2 2018 (4) | Q3 2015 | Q1 2017 |
| **CA** | Q4 2016 (2) | Q1 2015 (2) | Q4 2019 (1) | Q4 2019 (3) | Q4 2015 | Q3 2017 |
| **FR** | Q4 2016 (2) | Q1 2015 (4) | Q4 2017 (1) | Q3 2018 (5) | Q2 2015 | Q1 2018 |
| **DE** | Q1 2016 (2) | Q1 2015 (4) | Q2 2017 (2) | Q2 2018 (5) | Q3 2015 | Q1 2017 |
| **IT** | Q4 2016 (2) | Q1 2015 (4) | Q3 2017 (2) | Q3 2018 (5) | Q4 2015 | Q2 2017 |
| **JP** | Q2 2018 (4) | Q4 2014 (5) | Q1 2018 (2) | Q3 2018 (4) | Q3 2014 | Q1 2017 |
| **KR** | Q4 2018 (2) | Q4 2012 (2) | Q3 2017 (1) | Q3 2017 (2) | Q4 2015 | Q2 2015 |
| **ES** | Q4 2016 (2) | Q1 2015 (4) | Q3 2017 (2) | Q3 2018 (5) | Q1 2016 | Q3 2017 |
| **SE** | Q1 2016 (2) | Q1 2015 (4) | Q1 2018 (2) | Q3 2018 (4) | Q3 2015 | Q1 2017 |
| **CH** | Q2 2018 (2) | Q1 2016 (2) | Q3 2018 (2) | Q4 2019 (2) | Q4 2015 | Q3 2017 |
| **UK** | Q1 2016 (3) | Q1 2015 (4) | Q2 2017 (3) | Q2 2018 (5) | Q3 2015 | Q1 2017 |

*AT* Austria, *AU* Australia, *CA* Canada, *CH* Switzerland, *DE* Germany, *ES* Spain, *FR* France, *IT* Italy, *JP* Japan, *KR* South Korea, *MIDAS* Multinational Integrated Data Analysis System, *Q1* first quarter, *Q2* second quarter, *Q3* third quarter, *Q4* fourth quarter, *SE* Sweden, *UK* United Kingdom

Group A = Molecules for which biosimilar data were available during the study period (etanercept, infliximab, rituximab, and trastuzumab), Group B = Molecules for which biosimilar data were not available during the study period (cetuximab, nivolumab, and pembrolizumab), Group A & cetuximab originators were entered the market before Q1 2011, for Group A, the number of marketed biosimilar products was tabulated at the brand-name level.

**Supplementary Table 3.** Group A and B weighted average prices in Q1 2018 (USD per SU, Selected dosage form only).

|  | **Group A** | | | | **Group B** | | |
| --- | --- | --- | --- | --- | --- | --- | --- |
|  | **Etanercept** | **Infliximab** | **Rituximab** | **Trastuzumab** | **Cetuximab** | **Nivolumab** | **Pembrolizumab** |
| **Selected dosage** | 50mg/ml*1ml (Korea 25mg/ml*1ml, 50mg/ml*0.5ml) | 100mg | 10mg/ml*50ml | 150mg (Canada 440mg) | 100mg (5mg/ml*20ml, 2mg/ml*50ml) | 10mg/ml*10ml | 25mg/ml*4ml |
| **AU** | 184.22 | 334.35 | 1447.56 | 769.40 | 260.93 | 1623.29 | 3509.08 |
| **AT** | 200.39 | 363.71 | 1642.86 | 847.92 | 244.12 | 1753.13 | 4212.55 |
| **CA** | 305.99 | 749.75 | 1820.12 | 2149.26 | 265.67 | 1503.49 | 3382.89 |
| **FR** | 194.07 | 430.82 | 1020.67 | 521.52 | 202.85 | 1273.02 | 3230.33 |
| **DE** | 386.00 | 577.27 | 1567.43 | 830.90 | 270.87 | 1225.05 | 3090.08 |
| **IT** | 256.82 | 499.35 | 1427.48 | 674.73 | 209.67 | 1651.60 | 4212.54 |
| **JP** | 226.21 | 591.82 | 1524.27 | 415.61 | 267.84 | 2647.41 | 2930.23 |
| **KR** | 65.54 | 289.35 | 802.52 | 321.58 | 174.69 | 1030.28 | 2221.15 |
| **ES** | 207.76 | 494.26 | 1476.38 | 733.04 | 236.31 | 1751.13 | 4382.13 |
| **SE** | 210.47 | 475.70 | 1414.58 | 685.10 | 249.30 | 1526.18 | 3925.34 |
| **CH** | 425.49 | 718.06 | 1652.62 | 668.74 | 255.29 | 1635.66 | 2530.11 |
| **UK** | 204.11 | 465.12 | 977.85 | 495.97 | 216.84 | 1335.46 | 3202.05 |

*AT* Austria, *AU* Australia, *CA* Canada, *CH* Switzerland, *DE* Germany, *ES* Spain, *FR* France, *IT* Italy, *JP* Japan, *KR* South Korea, *Q1* first quarter, *Q2* second quarter, *Q3* third quarter, *Q4* fourth quarter, *SE* Sweden, *SU* Standard Unit, *USD* United States Dollar, *UK* United Kingdom

Group A = Molecules for which biosimilar data were available during the study period (etanercept, infliximab, rituximab, and trastuzumab), Group B = Molecules for which biosimilar data were not available during the study period (cetuximab, nivolumab, and pembrolizumab).

**Supplementary Table 4.** Group A and B total expenditure trends, Q1 2018–Q2 2020 (million USD, Selected dosage from only).

|  | **Group A** | | | | | | | | | | | **Group B** | | | | | | | | | | |
| --- | --- | --- | --- | --- | --- | --- | --- | --- | --- | --- | --- | --- | --- | --- | --- | --- | --- | --- | --- | --- | --- | --- |
|  | **Q1**  **2018** | **Q2**  **2018** | **Q3**  **2018** | **Q4**  **2018** | **Q1**  **2019** | **Q2**  **2019** | **Q3**  **2019** | **Q4**  **2019** | **Q1**  **2020** | **Q2**  **2020** | **Q1**  **2018** | | **Q2**  **2018** | **Q3**  **2018** | **Q4**  **2018** | **Q1**  **2019** | **Q2**  **2019** | **Q3**  **2019** | **Q4**  **2019** | **Q1**  **2020** | **Q2**  **2020** |  |
| **AU** | 51.24 | 49.05 | 45.94 | 45.66 | 41.84 | 42.12 | 39.57 | 35.98 | 33.33 | 28.46 | 25.51 | | 23.46 | 21.66 | 22.61 | 23.83 | 24.88 | 27.72 | 29.45 | 32.43 | 35.23 |  |
| **AT** | 28.01 | 28.27 | 28.78 | 29.92 | 26.07 | 24.39 | 22.59 | 21.03 | 19.93 | 18.13 | 24.42 | | 25.96 | 25.34 | 24.90 | 28.83 | 32.69 | 36.84 | 41.74 | 43.72 | 45.90 |  |
| **CA** | 345.21 | 354.64 | 346.39 | 350.66 | 331.77 | 339.23 | 353.03 | 349.91 | 345.01 | 322.07 | 28.56 | | 38.93 | 46.91 | 52.99 | 50.53 | 61.72 | 73.12 | 96.73 | 93.01 | 99.93 |  |
| **FR** | 238.81 | 206.59 | 201.83 | 192.24 | 185.20 | 187.15 | 192.06 | 205.04 | 156.55 | 148.79 | 139.45 | | 144.95 | 170.82 | 190.58 | 200.32 | 210.96 | 143.17 | 152.16 | 174.99 | 181.90 |  |
| **DE** | 364.33 | 348.87 | 323.68 | 310.49 | 290.70 | 271.23 | 274.80 | 246.82 | 240.32 | 215.28 | 86.76 | | 97.10 | 108.94 | 119.39 | 139.93 | 181.44 | 217.91 | 217.67 | 231.56 | 233.22 |  |
| **IT** | 169.40 | 167.21 | 161.03 | 155.88 | 145.63 | 145.75 | 142.04 | 136.92 | 139.86 | 131.46 | 140.98 | | 160.06 | 166.78 | 180.81 | 189.82 | 211.99 | 209.77 | 207.72 | 233.98 | 228.25 |  |
| **JP** | 291.93 | 297.41 | 283.60 | 287.59 | 257.96 | 273.13 | 279.88 | 273.12 | 235.81 | 245.70 | 296.23 | | 306.87 | 312.93 | 329.44 | 236.57 | 264.57 | 311.62 | 284.64 | 262.55 | 237.28 |  |
| **KR** | 27.22 | 26.58 | 25.69 | 25.80 | 25.96 | 25.47 | 25.77 | 26.75 | 25.85 | 23.83 | 29.27 | | 30.59 | 33.65 | 39.18 | 41.70 | 45.07 | 44.89 | 47.57 | 45.57 | 45.75 |  |
| **ES** | 151.03 | 155.66 | 146.97 | 152.31 | 151.26 | 154.98 | 154.39 | 153.25 | 155.63 | 141.77 | 90.89 | | 101.47 | 102.45 | 110.47 | 113.44 | 123.83 | 138.07 | 152.30 | 161.77 | 162.50 |  |
| **SE** | 55.90 | 54.87 | 50.74 | 52.91 | 53.27 | 57.36 | 54.27 | 51.44 | 48.18 | 39.40 | 13.66 | | 14.66 | 14.18 | 10.54 | 12.52 | 13.89 | 15.13 | 16.32 | 17.96 | 24.85 |  |
| **CH** | 49.04 | 47.28 | 43.88 | 44.71 | 42.44 | 43.61 | 44.20 | 45.23 | 44.88 | 42.96 | 22.17 | | 26.33 | 29.53 | 32.37 | 24.09 | 25.09 | 26.34 | 29.35 | 31.79 | 32.55 |  |
| **UK** | 183.81 | 181.92 | 178.46 | 185.52 | 186.42 | 186.86 | 186.23 | 198.11 | 196.49 | 181.72 | 35.79 | | 39.85 | 41.14 | 46.76 | 55.25 | 63.41 | 82.95 | 95.28 | 98.41 | 87.30 |  |

*AT* Austria, *AU* Australia, *CA* Canada, *CH* Switzerland, *DE* Germany, *ES* Spain, *FR* France, *IT* Italy, *JP* Japan, *KR* South Korea, *Q1* first quarter, *Q2* second quarter, *Q3* third quarter, *Q4* fourth quarter, *SE* Sweden, *USD* United States Dollar, *UK* United Kingdom

Group A = Molecules for which biosimilar data were available during the study period (etanercept, infliximab, rituximab, and trastuzumab), Group B = Molecules for which biosimilar data were not available during the study period (cetuximab, nivolumab, and pembrolizumab), selected dosage form included cetuximab 100mg (5mg/ml*20ml, 2mg/ml*50ml), etanercept 50mg/ml*1ml (Korea 25mg/ml*1ml, 50mg/ml*0.5ml), infliximab 100mg, nivolumab 10mg/ml*10ml, pembrolizumab 25mg/ml*4ml, rituximab 10mg/ml*50ml, and trastuzumab 150mg (Canada 440mg).

**Supplementary Table 5.** Timing of supply- and demand-side policy implementation for biosimilars in 12 countries [Adapted from Copyright © 2025 (Shin et al, 2025)]

| **Policies for Biosimilars** | | **Countries** | | | | | | | | | | | | **References** | |
| --- | --- | --- | --- | --- | --- | --- | --- | --- | --- | --- | --- | --- | --- | --- | --- |
|  |  | **AU** | **AT** | **CA** | **FR** | **DE** | **IT** | **JP** | **KR** | **ES** | **SE** | **CH** | **UK** |  |  |
| **Supply-side** | **Price linkage** | 2017 ^a^ | 2014 ^a^ | N/A | 2015 | N/A | 2015^a^ | 2019^a^ | 2019 | 2016^a^ | N/A | 2017 ^a^ | N/A | (1-12) |  |
|  | **Tendering** | 2013 ^a^ | 2017 ^a^ | N/A | 2015^a^ | 2015^a^ | 2015^a^ | N/A | 2017^b^ | 2015^a^ | 2015 | 2020 ^a^ | 2015 | (1-7, 13-15) |  |
| **Demand-side** | **Financial incentives** | 2018 ^a^ | 2017 ^a^ | N/A | 2017^a^ | 2015 | 2015^a^ | 2021 | N/A | 2017^a, b^ | 2014^a^ | 2021 ^a^ | 2015 | (1-6, 16-18) |  |
|  | **Prescribing guidelines** | 2017 ^a^ | 2005^c^ | 2023 | 2015^a^ | 2017^a^ | 2016^a^ | N/A | N/A | 2015 | 2015 | 2021 ^a^ | 2015 | (2-6, 18-23) |  |
|  | **Prescription budget** | N/A | N/A | N/A | N/A | 2014^b^ | 2015^a^ | N/A | N/A | 2017^a, b^ | 2017^a^ | N/A | 2015 | (2-4, 24) |  |
|  | **Prescription quota** | N/A | N/A | N/A | 2017^b^ | 2015^b^ | 2015^a^ | N/A | N/A | 2016^a, b^ | 2017^a^ | N/A | N/A | (2-4, 25-27) |  |
|  | **Information and education** | 2015 | 2020 ^a^ | 2012 ^a^ | 2015^a^ | 2015 | 2015 | 2018^b^ | N/A | 2016 | 2015 | 2017 ^a^ | 2015 | (3, 4, 7, 15, 18, 28-30) |  |

*AT* Austria, *AU* Australia, *CA* Canada, *CH* Switzerland, *DE* Germany, *ES* Spain, *FR* France, *IT* Italy, *JP* Japan, *KR* South Korea, *Q1* first quarter, *Q2* second quarter, *Q3* third quarter, *Q4* fourth quarter, *SE* Sweden, *SU* Standard Unit, *USD* United States Dollar, *UK* United Kingdom

Adapted from Shin, G., Han, H., Choi, G., Lee, D., & Bae, S. (2025). Demand-Versus Supply-Side Policies in Market Penetration of Biosimilars: Which is More Effective?. *BioDrugs* 39, 635-644

^a^If the exact start date was unclear, it was confirmed that the policy had been in effect by at least the stated year

^b^Partially applied or indirectly implemented policies

^c^ The‘Guidelines on the economic prescription of medicines and therapeutic aids’ recommend that physicians prescribe the lowest-cost option among therapeutically equivalent products, including biosimilars.

*References:

1. Moorkens E, Vulto AG, Huys I, Dylst P, Godman B, Keuerleber S, Claus B, Dimitrova M, Petrova G, Sović-Brkičić L. Policies for biosimilar uptake in Europe: an overview. PloS one. 2017;12(12):e0190147. doi:https://doi.org/10.1371/journal.pone.0190147.

2. Machado S, Cruz A, Ferreira PL, Morais C, Pimenta RE. Policy measures and instruments used in European countries to increase biosimilar uptake: a systematic review. Frontiers in Public Health. 2024;12:1263472.

3. Kim Y, Kwon H-Y, Godman B, Moorkens E, Simoens S, Bae S. Uptake of biosimilar infliximab in the UK, France, Japan, and Korea: budget savings or market expansion across countries? Frontiers in pharmacology. 2020;11:970. doi:https://doi.org/10.3389/fphar.2020.00970.

4. Rémuzat C, Kapuśniak A, Caban A, Ionescu D, Radière G, Mendoza C, Toumi M. Supply-side and demand-side policies for biosimilars: an overview in 10 European member states. Journal of Market Access & Health Policy. 2017;5(1):1307315.

5. Vogler S, Schneider P, Zuba M, Busse R, Panteli D. Policies to encourage the use of biosimilars in European countries and their potential impact on pharmaceutical expenditure. Frontiers in pharmacology. 2021;12:625296. doi:https://doi.org/10.3389/fphar.2021.625296.

6. Alnaqbi KA, Bellanger A, Brill A, Castañeda-Hernández G, Clopés Estela A, Delgado Sánchez O, García-Alfonso P, Gyger P, Heinrich D, Hezard G. An international comparative analysis and roadmap to sustainable biosimilar markets. Frontiers in Pharmacology. 2023;14:1188368. doi:https://doi.org/10.3389/fphar.2023.1188368.

7. Piedade D, Verhelst H. 2020 Market Review - European Biosimilar Medicine Markets - Policy Overview. 2021. The Biosimilar Medicines Group - Market Access Committee.

8. Gregory GP, Carrington C, Cheah CY, Hawkes EA, Irving IM, Siderov J, Opat S. A consensus statement on the use of biosimilar medicines in hematology in Australia. Asia‐Pacific Journal of Clinical Oncology. 2020;16(4):211-221.

9. Biosimilar uptake drivers. Australian Government—Department of Health; 20 Sep 2025. Available from: http://www.pbs.gov.au/info/general/biosimilars.

10. Mestre-Ferrandiz J, Towse A, Berdud M. Biosimilars: how can payers get long-term savings? Pharmacoeconomics. 2016;34(6):609-616. doi:https://doi.org/10.1007/s40273-015-0380-x.

11. Carl DL, Laube Y, Serra-Burriel M, Naci H, Ludwig W-D, Vokinger KN. Comparison of Uptake and Prices of Biosimilars in the US, Germany, and Switzerland. JAMA network open. 2022;5(12):e2244670-e2244670. doi:https://doi.org/10.1001/jamanetworkopen.2022.44670.

12. Dranitsaris G, Jacobs I, Kirchhoff C, Popovian R, Shane LG. Drug tendering: drug supply and shortage implications for the uptake of biosimilars. ClinicoEconomics and Outcomes Research. 2017:573-584. doi:https://doi.org/10.2147/ceor.s140063.

13. Verghese NR, Barrenetxea J, Bhargava Y, Agrawal S, Finkelstein EA. Government pharmaceutical pricing strategies in the Asia-Pacific region: an overview. Journal of market access & health policy. 2019;7(1):1601060.

14. Power D. Licensing and prescribing biosimilars in Australia. Generics Biosimilars Initiat J. 2013;2(3):152-154. doi:https://doi.org/10.5639/gabij.2013.0203.030.

15. Dylst P. 2017 Market Review - European Biosimilar Medicine Markets - Policy Overview. 2017.

16. Moorkens E, Vulto AG, Kent J, McClure L, Boldero R, Vanhove T, Simoens S, Huys I. A look at the history of biosimilar adoption: Characteristics of early and late adopters of infliximab and etanercept biosimilars in subregions of england, scotland and wales-a mixed methods study. BioDrugs. 2021;35:75-87.

17. Jommi C, Bertolani A. PBI35 local policies on biosimilars: are they designed to optimize use of freed resources? Value in Health. 2020;23:S416. doi:https://doi.org/10.1016/j.jval.2020.08.113.

18. Pant S, Léséleuc Ld, Spry C. International policies on the appropriate use of biosimilar drugs. CADTH; 2018. (CADTH Environmental Scan).

19. läkemedel. B. LAKEMEDELSVERKET. . 2022 14 Mar 2025. Available from: https://www.lakemedelsverket.se/sv/tillstand-godkannande-och-kontroll/tillverkningstillstand/biologiska-lakemedel/biosimilarer#hmainbody4. Swedish.

20. Guidance on the licensing of biosimilar products. Medicines and Healthcare products Regulatory Agency; 2021 20 Sep 2025. Available from: https://www.gov.uk/government/publications/guidance-on-the-licensing-of-biosimilar-products#:~:text=The%20purpose%20of%20this%20guideline%20is%20to.

21. Biosimilars 2. Auflage, Version 1.0 Januar 2021 - Leitfaden der Arzneimittelkommission der deutschen Ärzteschaft (AkdÄ). (AkdÄ) AddÄ; 2021 20 Sep 2025. Available from: https://www.akdae.de/arzneimitteltherapie/lf/biosimilars. German.

22. Abitbol A, Chu L. What do the guidelines say about use of biosimilar insulin therapy? Simple practical considerations to guide clinicians in different patient subgroups—Sharing Canadian perspectives. Diabetes, Obesity and Metabolism. 2025. doi:https://doi.org/10.1111/dom.16278.

23. Vogler S, Haasis MA, Zimmermann N. PPRI Pharma Brief: Austria 2023. 2024. Austrian Federal Ministry of Social Affairs, Health, Care and Consumer Protection.

24. Renwick MJ, Smolina K, Gladstone EJ, Weymann D, Morgan SG. Postmarket policy considerations for biosimilar oncology drugs. The Lancet Oncology. 2016;17(1):e31-e38. doi:https://doi.org/10.1016/s1470-2045(15)00381-2.

25. Moorkens E, Barcina Lacosta T, Vulto AG, Schulz M, Gradl G, Enners S, Selke G, Huys I, Simoens S. Learnings from regional market dynamics of originator and biosimilar infliximab and etanercept in Germany. Pharmaceuticals. 2020;13(10):324.

26. les médicaments biosimilaire. Santé HAd; 2017 20 Sep 2025. Available from: https://www.has-sante.fr/jcms/c_2807411/fr/les-medicaments-biosimilaires.

27. Nagai S. Current situation of oncology biosimilars in Japan. The Lancet Oncology. 2021;22(3):e82. doi:https://doi.org/10.1016/s1470-2045(21)00029-2.

28. Moorkens E, Simoens S, Troein P, Declerck P, Vulto AG, Huys I. Different policy measures and practices between Swedish counties influence market dynamics: part 1—biosimilar and originator infliximab in the hospital setting. BioDrugs. 2019;33:285-297.

29. Marechal-Jamil J, Graf M, Pacheco A. 2023 Market Review - European Biosimilar Medicine Markets - Policy Overview. 2023. The Biosimilar Medicines Group Market Access Committee.

30. Kay J, Feagan BG, Guirguis MS, Keystone EC, Klein AV, Lubiniecki AS, Mould DR, Nyarko KA, Ridgway AA, Trudeau ME. Health Canada/BIOTECanada Summit on regulatory and clinical topics related to subsequent entry biologics (biosimilars), Ottawa, Canada, 14 May 2012. Biologicals. 2012;40(6):517-527. doi:https://doi.org/10.1016/j.biologicals.2012.09.010.

**Supplementary Table 6.** Fisher price index trends, Q1 2018-Q2 2020 (Q1 2018 in each country = 1.00, selected dosage form only).

|  | **Group A** | | | | | | | | | | | | **Group B** | | | | | | | | | | | |
| --- | --- | --- | --- | --- | --- | --- | --- | --- | --- | --- | --- | --- | --- | --- | --- | --- | --- | --- | --- | --- | --- | --- | --- | --- |
|  | **Q1**  **2018** | **Q2**  **2018** | **Q3**  **2018** | **Q4**  **2018** | **Q1**  **2019** | **Q2**  **2019** | **Q3**  **2019** | **Q4**  **2019** | **Q1**  **2020** | **Q2**  **2020** | **CQGR** | **Q1**  **2018** | | **Q2**  **2018** | **Q3**  **2018** | **Q4**  **2018** | **Q1**  **2019** | **Q2**  **2019** | **Q3**  **2019** | **Q4**  **2019** | **Q1**  **2020** | **Q2**  **2020** | **CQGR** |  |
| **AU** | 1.00 | 0.97 | 0.92 | 0.90 | 0.89 | 0.88 | 0.82 | 0.71 | 0.69 | 0.66 | -4.54% | 1.00 | | 0.99 | 0.99 | 0.99 | 0.99 | 0.99 | 0.99 | 0.98 | 0.97 | 0.97 | -0.36% |  |
| **AT** | 1.00 | 0.97 | 0.92 | 0.90 | 0.89 | 0.89 | 0.86 | 0.84 | 0.81 | 0.80 | -2.40% | 1.00 | | 1.00 | 1.00 | 1.00 | 1.00 | 1.00 | 1.00 | 1.00 | 1.00 | 1.00 | 0.01% |  |
| **CA** | 1.00 | 1.00 | 0.99 | 0.99 | 0.98 | 0.98 | 0.97 | 0.96 | 0.96 | 0.93 | -0.81% | 1.00 | | 1.00 | 1.00 | 1.00 | 0.99 | 0.99 | 1.00 | 1.00 | 1.01 | 1.01 | 0.09% |  |
| **FR** | 1.00 | 0.92 | 0.91 | 0.90 | 0.89 | 0.89 | 0.89 | 0.89 | 0.72 | 0.71 | -3.78% | 1.00 | | 1.00 | 1.00 | 1.00 | 1.00 | 0.99 | 0.99 | 0.99 | 0.98 | 0.98 | -0.20% |  |
| **DE** | 1.00 | 0.95 | 0.92 | 0.90 | 0.87 | 0.84 | 0.83 | 0.75 | 0.74 | 0.72 | -3.52% | 1.00 | | 1.01 | 1.01 | 0.98 | 0.98 | 0.98 | 0.98 | 0.98 | 0.98 | 0.98 | -0.19% |  |
| **IT** | 1.00 | 0.98 | 0.96 | 0.95 | 0.93 | 0.91 | 0.90 | 0.89 | 0.89 | 0.88 | -1.38% | 1.00 | | 1.00 | 1.00 | 1.00 | 1.00 | 1.00 | 1.00 | 1.00 | 1.00 | 1.00 | 0.00% |  |
| **JP** | 1.00 | 0.89 | 0.88 | 0.88 | 0.87 | 0.86 | 0.85 | 0.81 | 0.79 | 0.75 | -3.22% | 1.00 | | 0.84 | 0.84 | 0.73 | 0.75 | 0.78 | 0.78 | 0.80 | 0.73 | 0.61 | -5.39% |  |
| **KR** | 1.00 | 1.00 | 0.99 | 0.99 | 0.98 | 0.97 | 0.97 | 0.96 | 0.93 | 0.91 | -1.03% | 1.00 | | 1.00 | 1.00 | 1.00 | 1.00 | 1.00 | 1.00 | 1.00 | 1.00 | 1.00 | -0.01% |  |
| **ES** | 1.00 | 1.00 | 0.99 | 0.99 | 0.96 | 0.96 | 0.95 | 0.91 | 0.91 | 0.91 | -0.99% | 1.00 | | 1.00 | 1.00 | 1.00 | 1.00 | 1.00 | 1.00 | 1.00 | 1.00 | 1.00 | 0.00% |  |
| **SE** | 1.00 | 0.97 | 0.96 | 0.96 | 0.93 | 0.92 | 0.91 | 0.87 | 0.82 | 0.78 | -2.69% | 1.00 | | 1.00 | 1.00 | 1.00 | 1.00 | 1.00 | 1.00 | 1.00 | 1.00 | 1.00 | 0.00% |  |
| **CH** | 1.00 | 0.97 | 0.92 | 0.89 | 0.88 | 0.88 | 0.87 | 0.86 | 0.86 | 0.86 | -1.68% | 1.00 | | 0.96 | 0.95 | 0.95 | 0.95 | 0.96 | 0.96 | 0.96 | 0.96 | 0.96 | -0.49% |  |
| **UK** | 1.00 | 1.00 | 0.99 | 0.99 | 0.99 | 0.99 | 0.98 | 0.98 | 0.98 | 0.98 | -0.19% | 1.00 | | 1.00 | 1.00 | 1.00 | 1.00 | 1.00 | 1.00 | 1.00 | 1.00 | 1.00 | 0.00% |  |
| **AVG** | 1.00 | 0.97 | 0.95 | 0.93 | 0.92 | 0.91 | 0.90 | 0.87 | 0.84 | 0.82 | -2.19% | 1.00 | | 0.98 | 0.98 | 0.97 | 0.97 | 0.97 | 0.97 | 0.97 | 0.97 | 0.96 | -0.54% |  |
| **SD** | 0.00 | 0.03 | 0.04 | 0.04 | 0.04 | 0.05 | 0.06 | 0.08 | 0.09 | 0.10 | 1.32% | 0.00 | | 0.04 | 0.05 | 0.07 | 0.07 | 0.06 | 0.06 | 0.05 | 0.07 | 0.11 | 1.47% |  |
| **MED** | 1.00 | 0.97 | 0.94 | 0.92 | 0.91 | 0.90 | 0.89 | 0.88 | 0.84 | 0.83 | -2.04% | 1.00 | | 1.00 | 1.00 | 1.00 | 1.00 | 1.00 | 1.00 | 1.00 | 1.00 | 1.00 | -0.01% |  |
| **IQR** | 0.00 | 0.03 | 0.07 | 0.09 | 0.08 | 0.08 | 0.10 | 0.10 | 0.14 | 0.17 | 2.28% | 0.00 | | 0.00 | 0.00 | 0.01 | 0.01 | 0.01 | 0.01 | 0.02 | 0.02 | 0.02 | 0.24% |  |
| **P** |  |  |  |  |  |  |  |  |  |  |  |  | |  |  |  |  |  |  |  |  |  | <0.001 |  |

*AT* Austria, *AU* Australia, *AVG* average, *CA* Canada, *CH* Switzerland, *CQGR* Compound Quarterly Growth Rate, *DE* Germany, *ES* Spain, *FR* France, *IQR* interquartile range, *IT* Italy, *JP* Japan, *KR* South Korea, *MED* median, *P* p-value, *Q1* first quarter, *Q2* second quarter, *Q3* third quarter, *Q4* fourth quarter, *SD* standard deviation, *SE* Sweden, *UK* United Kingdom

Group A = Molecules for which biosimilar data were available during the study period (etanercept, infliximab, rituximab, and trastuzumab), Group B = Molecules for which biosimilar data were not available during the study period (cetuximab, nivolumab, and pembrolizumab), selected dosage form included cetuximab 100mg (5mg/ml*20ml, 2mg/ml*50ml), etanercept 50mg/ml*1ml (Korea 25mg/ml*1ml, 50mg/ml*0.5ml), infliximab 100mg, nivolumab 10mg/ml*10ml, pembrolizumab 25mg/ml*4ml, rituximab 10mg/ml*50ml, and trastuzumab 150mg (Canada 440mg), P-value was calculated by a Wilcoxon rank sum test.

**Supplementary Table 7.** Relative expenditure trends, Q1 2018-Q2 2020 (Q1 2018 in each country = 1.00, selected dosage from only).

|  | **Group A** | | | | | | | | | | | | **Group B** | | | | | | | | | | | |
| --- | --- | --- | --- | --- | --- | --- | --- | --- | --- | --- | --- | --- | --- | --- | --- | --- | --- | --- | --- | --- | --- | --- | --- | --- |
|  | **Q1**  **2018** | **Q2**  **2018** | **Q3**  **2018** | **Q4**  **2018** | **Q1**  **2019** | **Q2**  **2019** | **Q3**  **2019** | **Q4**  **2019** | **Q1**  **2020** | **Q2**  **2020** | **CQGR** | **Q1**  **2018** | | **Q2**  **2018** | **Q3**  **2018** | **Q4**  **2018** | **Q1**  **2019** | **Q2**  **2019** | **Q3**  **2019** | **Q4**  **2019** | **Q1**  **2020** | **Q2**  **2020** | **CQGR** |  |
| **AU** | 1.00 | 0.99 | 0.96 | 0.98 | 0.90 | 0.92 | 0.89 | 0.81 | 0.78 | 0.66 | -4.44% | 1.00 | | 0.96 | 0.91 | 0.97 | 1.03 | 1.09 | 1.25 | 1.33 | 1.52 | 1.65 | 5.73% |  |
| **AT** | 1.00 | 1.04 | 1.09 | 1.15 | 1.01 | 0.95 | 0.89 | 0.83 | 0.79 | 0.72 | -3.55% | 1.00 | | 1.10 | 1.10 | 1.10 | 1.28 | 1.46 | 1.67 | 1.90 | 1.99 | 2.10 | 8.58% |  |
| **CA** | 1.00 | 1.05 | 1.04 | 1.06 | 1.01 | 1.04 | 1.07 | 1.06 | 1.06 | 1.02 | 0.25% | 1.00 | | 1.39 | 1.70 | 1.94 | 1.86 | 2.29 | 2.67 | 3.54 | 3.46 | 3.83 | 16.11% |  |
| **FR** | 1.00 | 0.89 | 0.89 | 0.87 | 0.84 | 0.86 | 0.89 | 0.95 | 0.73 | 0.70 | -3.96% | 1.00 | | 1.07 | 1.29 | 1.47 | 1.55 | 1.65 | 1.13 | 1.21 | 1.40 | 1.46 | 4.26% |  |
| **DE** | 1.00 | 0.99 | 0.94 | 0.92 | 0.86 | 0.81 | 0.83 | 0.75 | 0.73 | 0.66 | -4.52% | 1.00 | | 1.15 | 1.33 | 1.48 | 1.74 | 2.29 | 2.78 | 2.78 | 2.97 | 3.00 | 12.98% |  |
| **IT** | 1.00 | 1.02 | 1.00 | 0.99 | 0.93 | 0.94 | 0.93 | 0.90 | 0.92 | 0.87 | -1.59% | 1.00 | | 1.17 | 1.25 | 1.38 | 1.46 | 1.64 | 1.64 | 1.64 | 1.85 | 1.81 | 6.79% |  |
| **JP** | 1.00 | 1.03 | 1.00 | 1.03 | 0.90 | 0.95 | 0.95 | 0.94 | 0.81 | 0.84 | -1.98% | 1.00 | | 1.04 | 1.09 | 1.16 | 0.81 | 0.91 | 1.04 | 0.96 | 0.89 | 0.80 | -2.52% |  |
| **KR** | 1.00 | 0.98 | 0.99 | 1.00 | 1.00 | 1.02 | 1.05 | 1.08 | 1.06 | 1.00 | -0.04% | 1.00 | | 1.05 | 1.20 | 1.41 | 1.50 | 1.68 | 1.71 | 1.78 | 1.73 | 1.78 | 6.61% |  |
| **ES** | 1.00 | 1.06 | 1.03 | 1.09 | 1.08 | 1.12 | 1.13 | 1.13 | 1.15 | 1.05 | 0.52% | 1.00 | | 1.15 | 1.19 | 1.31 | 1.35 | 1.49 | 1.68 | 1.86 | 1.98 | 2.00 | 7.98% |  |
| **SE** | 1.00 | 1.05 | 1.00 | 1.06 | 1.08 | 1.19 | 1.15 | 1.09 | 1.03 | 0.84 | -1.91% | 1.00 | | 1.15 | 1.14 | 0.86 | 1.04 | 1.18 | 1.31 | 1.42 | 1.57 | 2.17 | 8.99% |  |
| **CH** | 1.00 | 1.00 | 0.93 | 0.96 | 0.91 | 0.94 | 0.94 | 0.96 | 0.93 | 0.89 | -1.28% | 1.00 | | 1.23 | 1.38 | 1.53 | 1.14 | 1.20 | 1.23 | 1.38 | 1.46 | 1.49 | 4.55% |  |
| **UK** | 1.00 | 1.01 | 1.04 | 1.09 | 1.08 | 1.10 | 1.14 | 1.17 | 1.16 | 1.11 | 1.14% | 1.00 | | 1.14 | 1.23 | 1.41 | 1.65 | 1.92 | 2.62 | 2.88 | 2.99 | 2.73 | 11.82% |  |
| **AVG** | 1.00 | 1.01 | 0.99 | 1.01 | 0.97 | 0.99 | 0.99 | 0.97 | 0.93 | 0.86 | -1.78% | 1.00 | | 1.13 | 1.23 | 1.34 | 1.37 | 1.57 | 1.73 | 1.89 | 1.98 | 2.07 | 7.66% |  |
| **SD** | 0.00 | 0.04 | 0.05 | 0.08 | 0.08 | 0.11 | 0.11 | 0.13 | 0.15 | 0.15 | 1.91% | 0.00 | | 0.10 | 0.18 | 0.27 | 0.31 | 0.42 | 0.60 | 0.75 | 0.73 | 0.77 | 4.56% |  |
| **MED** | 1.00 | 1.01 | 1.00 | 1.01 | 0.97 | 0.95 | 0.94 | 0.96 | 0.93 | 0.85 | -1.75% | 1.00 | | 1.14 | 1.22 | 1.39 | 1.40 | 1.57 | 1.66 | 1.71 | 1.79 | 1.90 | 7.39% |  |
| **IQR** | 0.00 | 0.05 | 0.07 | 0.10 | 0.13 | 0.12 | 0.19 | 0.20 | 0.27 | 0.29 | 3.68% | 0.00 | | 0.09 | 0.17 | 0.33 | 0.46 | 0.54 | 0.69 | 0.75 | 0.74 | 0.70 | 4.26% |  |
| **P** |  |  |  |  |  |  |  |  |  |  |  |  | |  |  |  |  |  |  |  |  |  | <0.001 |  |

*AT* Austria, *AU* Australia, *AVG* average, *CA* Canada, *CH* Switzerland, *CQGR* Compound Quarterly Growth Rate, *DE* Germany, *ES* Spain, *FR* France, *IQR* interquartile range, *IT* Italy, *JP* Japan, *KR* South Korea, *MED* median, *P* p-value, *Q1* first quarter, *Q2* second quarter, *Q3* third quarter, *Q4* fourth quarter, *SD* standard deviation, *SE* Sweden, *UK* United Kingdom

Group A = Molecules for which biosimilar data were available during the study period (etanercept, infliximab, rituximab, and trastuzumab), Group B = Molecules for which biosimilar data were not available during the study period (cetuximab, nivolumab, and pembrolizumab), selected dosage form included cetuximab 100mg (5mg/ml*20ml, 2mg/ml*50ml), etanercept 50mg/ml*1ml (Korea 25mg/ml*1ml, 50mg/ml*0.5ml), infliximab 100mg, nivolumab 10mg/ml*10ml, pembrolizumab 25mg/ml*4ml, rituximab 10mg/ml*50ml, and trastuzumab 150mg (Canada 440mg), P-value was calculated by a two-sample t-test.

**Supplementary Table 8.** Two-way fixed effects regression results on relative expenditure and price index.

| **Variable** | **Main TWFE (ln Rel_exp)** | **TWFE + A×Policy** | **Alt DV: ln(Price index)** |
| --- | --- | --- | --- |
| **Group A (biosimilar)** | –0.452** (0.130) | –0.722 (0.461) | –0.043 (0.041) |
| **Price linkage** | –0.143 (0.142) | –0.372 (0.209) | –0.018 (0.042) |
| **Tendering** | –0.077 (0.043) | –0.055 (0.149) | 0.041* (0.017) |
| **Education** | –0.044 (0.042) | 0.082 (0.053) | 0.013 (0.018) |
| **ln(Total SU)** | 0.030 (0.057) | 0.244 (0.152) | –0.014 (0.018) |
| **A×Price linkage** | — | 0.477 (0.281) | — |
| **A×Tendering** | — | –0.014 (0.266) | — |
| **A×Financial incentives** | — | –0.187 (0.248) | — |
| **A×Guidelines** | — | –0.271 (0.310) | — |
| **A×Budget** | — | 0.221 (0.171) | — |
| **A×Quota** | — | 0.114 (0.108) | — |
| **A×Education** | — | –0.258+ (0.129) | — |
| **Observations** | 240 | 240 | 240 |
| **R²** | 0.607 | 0.663 | 0.622 |
| **Fixed effects** | Country, Quarter | Country, Quarter | Country, Quarter |

Coefficients are reported with cluster-robust SEs (in parentheses). *** p < 0.05, ** p < 0.01, *** p < 0.001**
Dependent variable: ln(Relative Expenditure) unless otherwise noted.

*Alt DV* Alternative Dependent Variable, *Rel_exp* relative expenditure, *SU* standard unit, *TWFE* Two-way fixed effects.


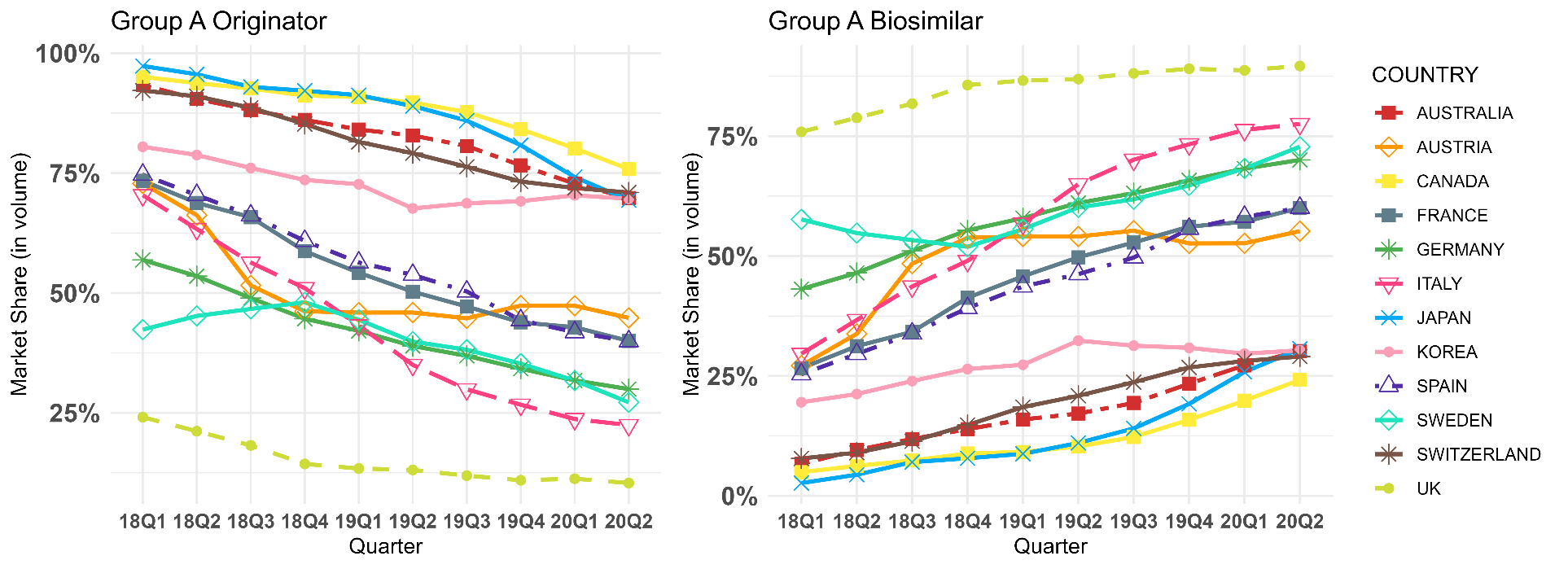


**Supplementary Figure 1.** Group A originator and biosimilar market share (in volume) trends in 12 countries, Q1 2018-Q2 2020 (selected dosage form only). Group A = Molecules for which biosimilar data were available during the study period (etanercept, infliximab, rituximab, and trastuzumab), selected dosage form included etanercept 50mg/ml*1ml (Korea 25mg/ml*1ml, 50mg/ml*0.5ml), infliximab 100mg, rituximab 10mg/ml*50ml, and trastuzumab 150mg (Canada 440mg).
